# Density Functional Calculation and Evaluation of the Spectroscopic Properties and Luminescent Material Application Potential of the N-Heterocyclic Platinum(II) Tetracarbene Complexes

**DOI:** 10.3390/molecules29020524

**Published:** 2024-01-20

**Authors:** Bao-Hui Xia, Yin-Si Ma, Fu-Quan Bai

**Affiliations:** 1 International Joint Research Laboratory of Nano-Micro Architecture Chemistry, College of Chemistry, Jilin University, Changchun 130023, China; 2 State Key Laboratory of Luminescent Materials and Devices, South China University of Technology, Guangzhou 510640, China

**Keywords:** potential energy curve, excited state, non-radiative deactivation, absorption and emission, platinum complex materials

## Abstract

A series of reported Pt(II) carbene complexes possibly have the ability to serve as the new generation of blue emitters in luminescent devices because of their narrow emission spectra, high photoluminescence quantum yields (PLQYs), and rigid molecular skeleton. However, the combination of all carbene ligands with different multidentate structures will affect the overall planarity and horizontal dipole ratio to varying degrees, but the specific extent of this effect has not previously been analyzed in detail. In this work, density functional computation is used to study a class of platinum tetracarbene bidentate complexes with similar absorption and emission band characteristics, which is the main reason for the remarkable difference in quantum efficiency due to subtle differences in electronic states caused by different ligands. From the calculation results, the major reason, which results in significantly decrease in quantum efficiency for [Pt(cyim)_2_]^2+^, is that [Pt(cyim)_2_]^2+^ can reach the non-radiative deactivation metal-centered d-d excited state through an easier pathway compared with [Pt(meim)_2_]^2+^. The result, based on changes in the dihedral angle between ligands, can achieve the goal of improving and designing materials by adjusting the degree of the dihedral angle. (meim: bis(1,1′-dimethyl-3,3′-methylene-diimidazoline-2,2′-diylidene); cyim: bis(1,1′-dicyclohexyl-3,3′-methylene-diimidazoline-2,2′-diylidene).

## 1. Introduction

Over the last decade, transition metals with N-heterocyclic carbenes (NHCs) as ligands have become the subject of intensive study in many fields of chemistry and molecular material design [1,2]. The bond between the C atom of N-heterocyclic carbene and the metal center of a complex in the molecular skeleton can be best described as a dative σ-bond when the M-C distance falls comfortably in the range of typical single M-C bond lengths and hybridized orbital components [3,4]. Their relative ease of synthesis from readily accessible precursors, together with their favorable donor properties, makes them the major choice for applications of the ligand [5,6,7].

**Figure 1:**
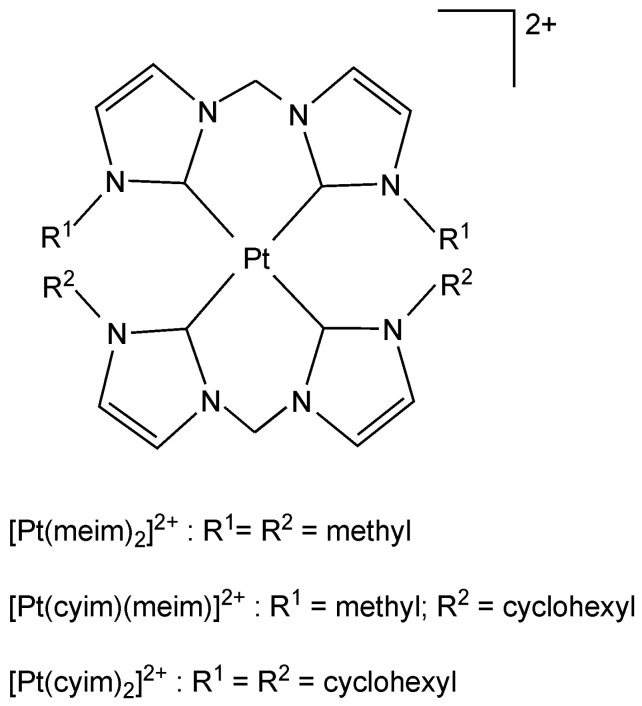
The structures of [Pt(meim)_2_]^2+^, [Pt(cyim)(meim)]^2+^ and [Pt(cyim)_2_]^2+^.

Our interest is the application of the transition metal NHC complexes in phosphorescent organic light-emitting devices (PhOLED) [8,9]. Surprisingly, the applications of platinum carbenes in PhOLED are rarely reported in the literature. Strassner and co-workers have reported the photophysical and photochemical properties of a series of platinum (II) complexes with biscarbene ligands (Figure 1) [10,11,12]. It is noted that these complexes show high photostability and can be used as blue phosphorescent emitters, of which substituents of N atoms are wavelength-independent. In addition, they exhibit not only an attractive quantum yield but also the quantum yield of Pt(meim)_2_^2+^ is seven times larger than that of Pt(cyim)_2_^2+^. (meim: bis(1,1′-dimethyl-3,3′-methylene-diimidazoline-2,2′-diylidene); cyim: bis(1,1′-dicyclohexyl-3,3′-methylene-diimidazoline-2,2′-diylidene).

This small difference in geometry leads to a large difference in the stability and inactivation characteristics of the excited state, which drives us to find the cause from the perspective of calculating the electronic structure of the excited state properties, and this factor is also applicable to other Pt-NHC-type complexes [13,14,15]. In coordination chemistry, the study of excited-state properties is a cornerstone for OLED luminescence phenomena. In this paper, a full-density functional theory (DFT) [16,17,18] and time-dependent density theory (TDDFT) [19,20] are used to investigate the geometry, electronic structures, and optical properties of Pt NHC complexes. This computational strategy has been repeatedly applied to transition metal complexes, which are metal-containing large-scale molecular systems, and the results are comparable to the experimental characterization. Therefore, they can provide a definitive characterization of the photophysical properties of this system and lead to deep understanding of this system.

## 2. Results and Discussion

**Figure 2:**
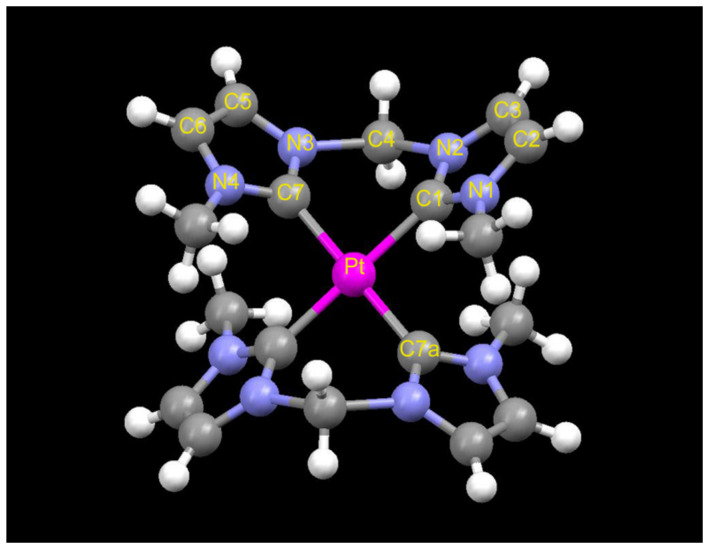
The optimized geometry of Pt(meim)_2_^2+^.

The optimized structural parameters of [Pt(meim)_2_]^2+^, [Pt(meim)(cyim)]^2+^, and [Pt(cyim)_2_]^2+^ are listed in Table 1. The metal atom and four carbon atoms on carbine are situated on the same plane, and substituents linking on N atoms are above or below the plane. Imidazole planes keep an inclination away from the metal carbene plane (Figure 2). The dihedral angle between the imidazole plane and the carbine plane decreases along with an increase in the bulk of the substituent.

**Figure 3:**
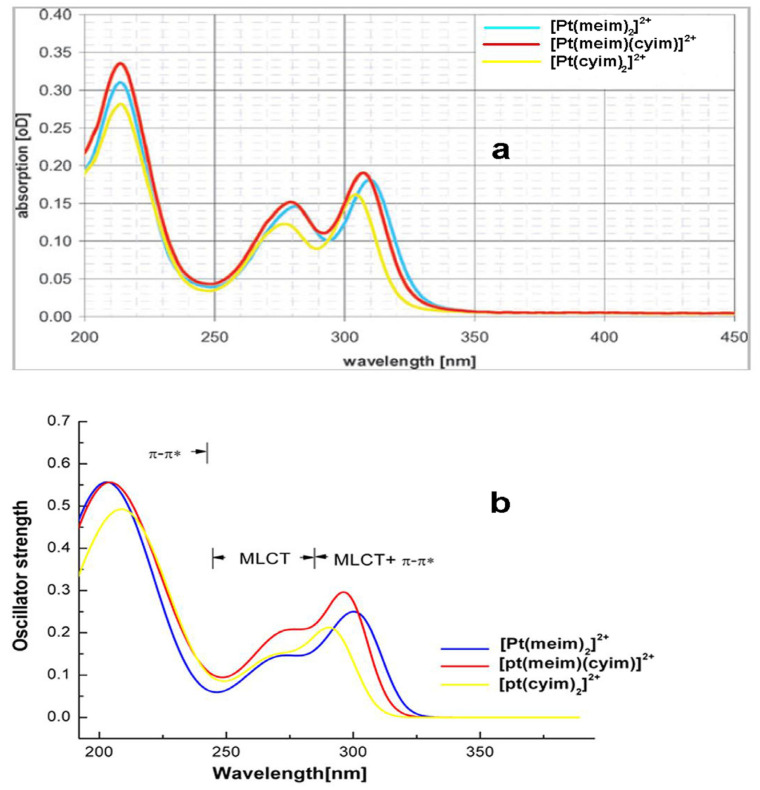
Simulated absorption spectra of topic complexes compared to the experimental spectra: (**a**) experimental absorption spectra obtained from Refs. [10,11], (**b**) calculated absorption spectra.

Figure 3 shows the simulated absorption spectra of these complexes. The experimental results [7] for three complexes (Figure 3a) are all reproduced well by the DFT calculations (Figure 3b). [Pt(meim)_2_]^2+^ has a lowest-energy absorption band around 300 nm, which can be assigned to MLCT/π → π* character. This transition is from HOMO to LUMO and from HOMO-1 to LUMO. The shoulder band at the region of 240–270 nm corresponds to the MLCT-type transition, which is dominated by the excitation from HOMO-3 to LUMO+1. The high-energy absorption bands are attributed to π → π* transition on the ligand. The absorption bands of [Pt(meim)(cyim)]^2+^ and [Pt(cyim)_2_]^2+^ are similar to those of [Pt(meim)_2_]^2+^. But MLCT transition bands of both complexes are blue shifted compared with [Pt(meim)_2_]^2+^, because the orbital energy change is affected by the decrease in the dihedral angle between the imidazole planes of the biscarbene ligand. The heteroleptic [Pt(meim)(cyim)]^2+^ has stronger oscillator strength in the lowest-energy absorption, which may be due to the low amount of LLCT character included in this absorption.

In the lowest-lying triplet excited state (T_1_) of these complexes (Table 1), bond distances of the Pt-C and C (carbene C)-N are significantly longer than those in the ground state (S_0_), and dihedral angles of C1–C4–Pt–C7 are more stretched. Differences in the bond length and the dihedral angle of T_1_ and S_0_ of [Pt(cyim)_2_]^2+^ are larger than those of [Pt(meim)_2_]^2+^. In [Pt(meim)(cyim)]^2+^, the expanded range of the big substituent side is larger than that of the other side. The ΔSCF energies of these complexes calculated from T_1_ geometries show similar values: 3.72 eV in [Pt(meim)_2_]^2+^, 3.84 eV in [Pt(cyim)_2_]^2+^, and 3.76 eV in [Pt(meim)(cyim)]^2+^, respectively. A better estimate of emission energies was obtained by TDDFT calculation at the T_1_ geometries. All three complexes display a low-lying state with ^3^MLCT character. [Pt(meim)_2_]^2+^, [Pt(cyim)_2_]^2+^, and [Pt(meim)(cyim)]^2+^ emit a band at 375 nm (3.31 eV), 369 nm (3.36 eV), and 372 nm (3.33 eV), respectively.

From the emission wavelength point of view, complexes in this class are good candidates for dopants in the deep blue region. However, the quantum yield of [Pt(cyim)_2_]^2+^ is fairly low ([Pt(meim)_2_]^2+^, at 0.45; for [Pt(meim)(cyim)]^2+^, it is 0.39, but for [Pt(cyim)_2_]^2+^, it is 0.06). PLQYs are determined by both the radiative decay rate constant (*k_r_*) and the non-radiative decay rate constant (*k_nr_*). It is a competitive process in general. Importantly, the *k_r_* from the T*_m_* state to the S_0_ state can be expressed as follows within the Born–Oppenheimer approximation and perturbation theory and frozen core calculation [15,21,22]:

(1) $$k_{r}^{\alpha }\left(T_{m}\to S_{0}\right)=\frac{\eta^{2}}{1.5}E{\left(T_{m}\right)}^{3}{\left\{\sum_{n}\frac{\langle T_{m}^{\alpha }|H_{SOC}|S_{n}\rangle }{E\left(S_{n}\right)-E\left(T_{m}\right)}{\left(\frac{f_{n}}{E\left(S_{n}\right)}\right)}^{1/2}\right\}}^{2}$$

(2) $$k_{r}=\frac{1}{3}\sum_{\alpha }k_{r}^{\alpha }$$

where *η* is the refractive index of the medium, and energy levels *E*(*T_m_*) and *E*(*S_n_*) are the energy of *m*th triplet excited state and *n*th singlet excited state, respectively. It is worth noting that the $\left\langle T_{m}^{\alpha }|H_{SOC}|S_{n}\right\rangle$ is the spin-orbit coupling (SOC) matrix elements between the *m*th triplet excited state and the *n*th singlet excited state, and the superscript *α* is the spin sublevel of the triplet excited state *T_m_* (*α* = x, y, or z). Finally, the *ƒ_n_* is the oscillator strength of the excited state transitions. Based on Equation (2), the value of *k_r_* is overall determined by the SOC matrix elements $\left\langle T_{m}^{\alpha }|H_{SOC}|S_{n}\right\rangle$, the oscillator strength *ƒ_n_*, and the energy gap between the coupled S*_n_* and T*_m_*. The calculated average *k_r_* values of the T_1_ → S_0_ transition [Equation (2)] are 7.72, 4.95, 5.83 × 10^5^ s^−1^ for [Pt(meim)_2_]^2+^, [Pt(meim)(cyim)]^2+^, and [Pt(cyim)_2_]^2+^, respectively. From the index of radiation deactivation rate of the low-lying ^3^MLCT excited state, there is little difference between the three complexes, indicating that the radiation deactivation path is not the key reason affecting the PLQYs. Extensive studies of transition metal complexes reveal that such thermal quenching of the emissive state can be ascribed to deactivation via a metal-centered (d-d) excited state [23,24,25,26]. The calculated geometrical parameters for the d-d state are given in Table 1. It indicates that this state is populated through the absorption of light, then the molecule undergoes a significant distortion upon the formation of the excited state and the increase in Pt-C bond lengths.

**Figure 4:**
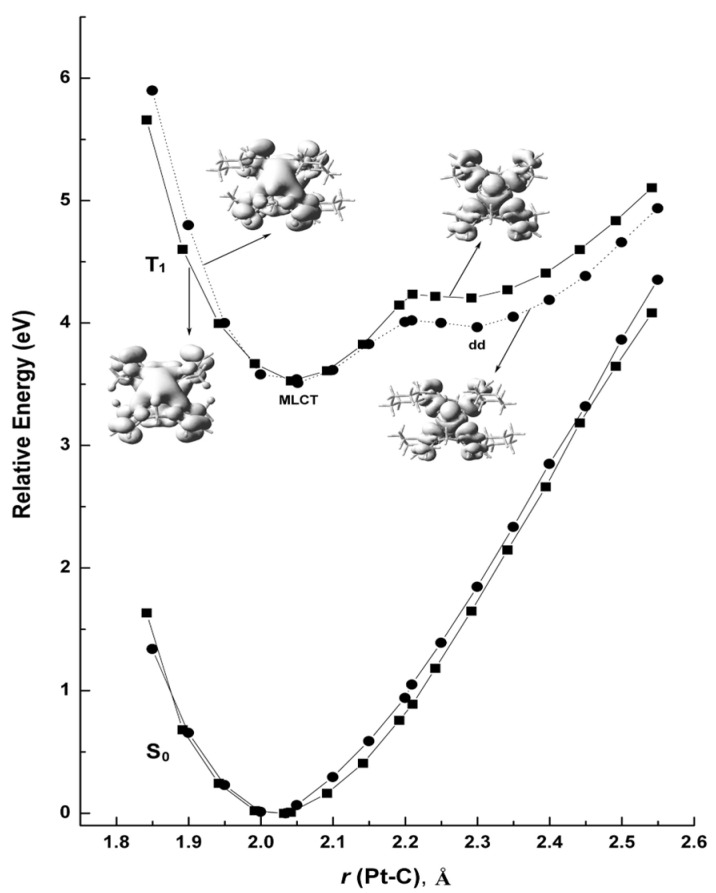
Potential energy curves of the ground state (S_0_) and the lowest triplet state (T_1_) involving the spin density (■: [Pt(meim)_2_]^2+^; ●: [Pt(cyim)_2_]^2+^).

**Figure 5:**
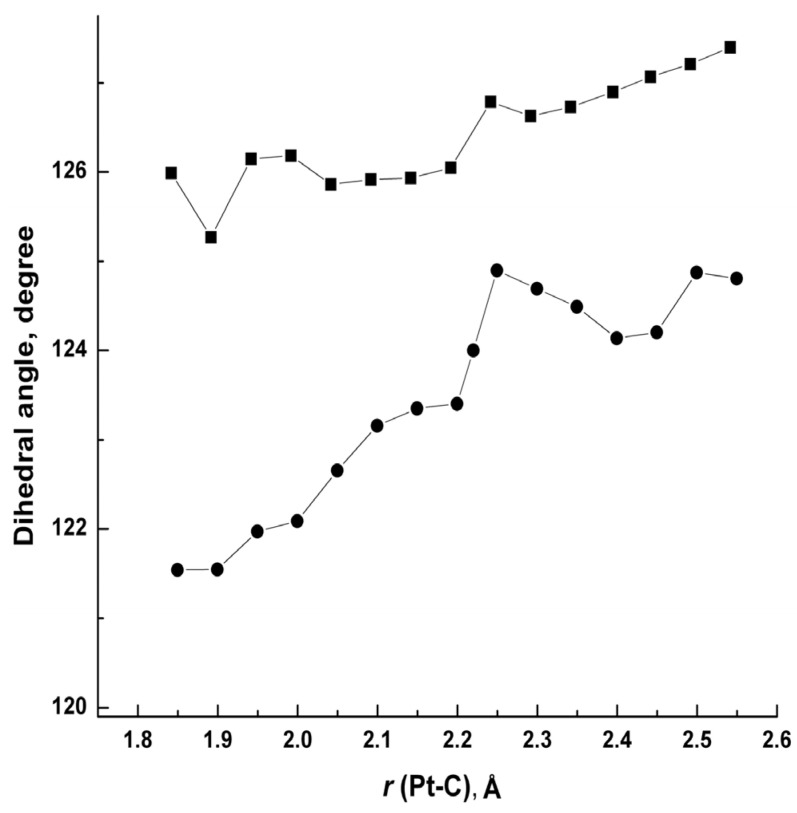
The change in dihedral angles of C1–C4–Pt–C7 in T_1_ (■: [Pt(meim)_2_]^2+^; ●: [Pt(cyim)_2_]^2+^).

To obtain insights into the deactivation process and analyze the vibration relaxation process of the phosphorescent state, the potential energy curves (PECs) [27,28,29,30,31,32] were calculated. Along with the symmetric stretching vibration route of the Pt-C bond ([Pt(meim)_2_]^2+^, 1121.77 cm^−1^; [Pt(cyim)_2_]^2+^, 1104.42 cm^−1^), the PECs were computed as a function of the Pt-C (carbene) distance. T_1_-PECs and S_0_-PECs are shown in Figure 4. The bond length of Pt-C in the lowest d-d state is considerably as long as 2.3 Å, while lengths and angles of other bonds are almost the same as those in the ground state. Dihedral angles between imidazole planes are outspread with structural relaxation (Figure 5). The change in dihedral angles in [Pt(cyim)_2_]^2+^ with bulky substituents is sharper than that of [Pt(meim)_2_]^2+^. As seen from Figure 5, when the Pt-C bond length extends over 2.2 Å, the dihedral angle has an abrupt increase, and the dihedral angle reaches the peak value when the lowest d-d state arrives.

The PECs for [Pt(meim)_2_]^2+^ and [Pt(cyim)_2_]^2+^ are illustrative for understanding the thermal deactivation of the phosphorescent states in transition metal complexes. There are three important reasons contributing to the reason that the d-d state of [Pt(cyim)_2_]^2+^ is induced more easily than that of [Pt(meim)_2_]^2+^. First, when the complex is excited, the resultant Franck–Condon state is relaxed into the T_1-MLCT_ potential minima. Under the fitting temperature, the excited state has sufficient thermal energy to go beyond the humps and reaches the d-d state. The activation energy barrier of [Pt(cyim)_2_]^2+^ is smaller than that in [Pt(meim)_2_]^2+^; the difference is about 0.11 eV in the T_1_ state. Second, the bond lengths of Pt-C for [Pt(cyim)_2_]^2+^ in T_1-MLCT_ are longer than that of [Pt(meim)_2_]^2+^, and in the d-d state, they have almost similar Pt-C bond lengths. Therefore, the geometry of [Pt(cyim)_2_]^2+^ undergoes less change (0.24 Å vs. 0.25 Å in [Pt(meim)_2_]^2+^) from T_1-MLCT_ to T_1-dd_. Finally, from the calculated reorganization energies [33,34,35], it is found that d-d states for both [Pt(meim)_2_]^2+^ and [Pt(cyim)_2_]^2+^ remain at a high level, and the energy level of [Pt(meim)_2_]^2+^ is higher than that of [Pt(cyim)_2_]^2+^, which is about 0.037 eV. In the excited state, the higher activation energy and larger structural difference upon vibration relaxation and the higher d-d state reorganization energy for [Pt(meim)_2_]^2+^ enhanced the excited state stability, so [Pt(meim)_2_]^2+^ has a good quantum yield. Comparing [Pt(cyim)_2_]^2+^ with [Pt(meim)_2_]^2+^, the bulky substituents induce a more out-of-plane deformation. From Table 2, we can see that the d orbital components of central metal in the frontier orbitals of [Pt(cyim)_2_]^2+^ are less than those in [Pt(meim)_2_]^2+^, and there is an obvious difference between d-d states. The increase in the twist angle of the carbene plane reduces the metal components in the frontier MOs and decreases the d-d state energy, which makes the d-d state easier to achieve. This situation could be avoided in future experiments.

We have also studied the charge transfer reorganization energies at the molecular level. In the case of solid-state optoelectronic devices, the external contribution reorganization energy (λ_e_) is negligible. Therefore, the inner reorganization energy (λ_i_) is approximate to the reorganization energy (λ). The reorganization energies of [Pt(meim)_2_]^2+^ and [Pt(cyim)_2_]^2+^ are listed in Table 2. For each molecule, reorganization energies for the hole transport are smaller than those of the electron transport, in general. This is determined by the fundamental characteristics of the particle of the electron and the hole. Compared with the classical benchmark of hole-transport material 4,4′-bis(phenyl-m-tolylamino)biphenyl (TPD) [36], of which λ_i(h)_ is 0.27 eV and λ_i(e)_ is 0.69 eV at the B3LYP/6-31G* level, both of the complexes have smaller λ_i(h)_. This means that the hole-transporting performance of these complexes is better than the electron-transporting performance.

The results presented in this work demonstrate that photophysical properties, especially the excited-state non-radiative deactivation pathways, can be strongly affected by different ligands. Access to such information, especially the structure-property relationships based on the characteristics of excited electronic states, is fundamental for a rational design of new metal complexes with tunable photochemical features. Photophysical measurements with emerging capabilities show advantages in investigating the excited-state potential energy surfaces. Hence, when combining photophysical measurements and quantum chemical calculations, it is helpful to provide deep information about such effects. This is also based on a precise understanding of electronic structural information and chemical bonds. The [Pt(meim)_2_]^2+^ and [Pt(cyim)_2_]^2+^ systems also have a large effect on the excitation state energy surface only because of the small difference in the M-C interaction caused by the coordination environment, which leads to a large difference in the non-radiative deactivation characteristics. The introduction of advantageous structural variations on photoactivable metal species can only be guided by the correct description of orbital energies and shapes, binding features, and a full comprehension holding of different excited states in light-caused electronic transitions.

## 3. Computational Details

All calculations here were performed with the Gaussian 09 program package [37], employing the various density functional theory (DFT) methods. Among Becke’s 3-parameter hybrid method, the long-range corrected functional CAM-B3LYP was set [38,39,40,41,42]. The effective core potential (ECP) basis set of the LanL2DZ [43,44,45] type with an additional f-polarization function (f = 0.18) for the transition metal platinum atom and 6-31G** basis set for the other atoms were used to optimize the ground state (S_0_) geometries [46,47]. To avoid the underestimation of the lowest-lying triplet state (T_1_) energy and some relaxation error in the optimized geometry [48] from time-dependent CAM-B3LYP calculations, the T_1_ geometries for the three complexes were calculated in the gas phase by using the unrestricted CAM-B3LYP functional. Based on the crystal data (distance Pt-I > 5 Å, the two iodide anions are non-coordinating), the calculation model corresponds to the complexes. By using the respective optimized equilibrium geometry, time-dependent density functional theory (TD-DFT) at the CAM-B3LYP level was employed and examined to predict their absorption and emission transitions and features. CAM-B3LYP yields atomization energies of similar quality to those from B3LYP while also performing well for charge transfer excitations, which B3LYP underestimates enormously. The ΔSCF approach [49] at the same level was also used for evaluating the emission energies of the complexes.

In order to determine the geometry of the metal-centered state (MC state or d-d state) [23], which is the most important state evidently involved in the deactivation process, preliminary geometrical optimizations were performed with constraints conditions based on strengthening M-C bonds. In detail, four metal-to-ligand bond lengths were elongated by 30% of the S_0_ geometry. It is found that most of the highest singly occupied orbitals are dσ* of the Pt-C bond in the resulting geometry. To obtain insights into the deactivation process and analyze the vibration relaxation process of the phosphorescent state, the potential energy curves (PECs) were established. Along with the symmetric stretching vibration route ([Pt(meim)_2_]^2+^, 1121.77 cm^−1^; [Pt(cyim)_2_]^2+^, 1104.42 cm^−1^), the PECs were computed as a function of the distance between Pt and the C atom of carbene by 0.05 Å increments. Hence, the Pt-C bond was frozen every 0.05 Å, and the geometry of the molecule was relaxed to a stationary point. From the view of the Franck–Condon principle, the S_0_-PECs were produced based on every T_1_ state geometry. That is to say, the PECs of the ground state have been emendated by the same method used in the excited state PECs calculation. Each stretching geometry was then employed to calculate the triplet excited state by TD-DFT at the CAM-B3LYP level. Except for the geometry optimization, the energy calculations in the PECs and TD-DFT calculations were performed with a large basis set. The 18-VE (valence electron) quasi-relativistic pseudo-potential and basis set of Andrae [50] with an additional f-polarization function (f = 0.14) are used for the Pt atom, and the 6-31G (3 df, 3 pd) basis set is used for all other atoms.

The Arrhenius equations:

*k*_d_(*T*) = *A*_1_ exp(−*E*_a1_/*kT*) + *A*_2_ exp(−*E*_a2_/*kT*)

where *k*_d_(*T*) is the temperature-dependent decay rate, and the pre-exponential *A*_1_ and energy level *E*_a1_ are the frequency factor and activation energy for the thermal deactivation through the higher-lying excited state, respectively. Similarly, the *A*_2_ and *E*_a2_ are those for the deactivation directly from the emitting state to the ground state contrastively.

**Figure 6:**
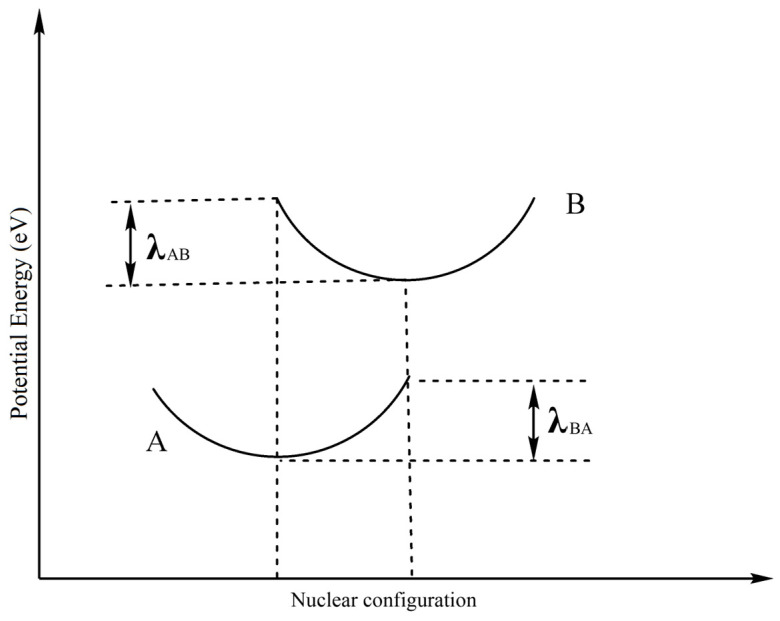
Schematic description of inner reorganization energy calculation between state A and B.

The rate of intermolecular charge transfer (*K*_et_) can be estimated by using the semiclassical MARCUS theory [51,52], which is described as follows:

*K*_et_ = *A* exp(−*λ*/4*K*_B_*T*)

where *A* is a prefactor related to the electronic coupling between adjacent molecules, and *λ* is the reorganization energy between the starting state and the final state. Classically, *K*_B_ is the Boltzmann constant, and *T* is the temperature. From this equation, it can be concluded that the reorganization energy is dominant in the charge transport process at constant temperature. Generally, the *λ* value is determined by fast changes in intramolecular geometry (the inner reorganization energy *λ*_i_) and slow variations in the molecular skeleton in solvent polarization of the surrounding medium (the external contribution *λ*_e_). However, the *λ*_e_ is negligible in most cases of solid-state optoelectronic devices, such as LEDs. Hence, the *λ*_i_ value is approximate to the total *λ*. Furthermore, the inner reorganization energy *λ*_i_ is caused by the change in the internal nuclear coordinates, which is from state A to state B (Figure 6), and the conventional reorganization energy *λ* is the sum of *λ*_AB_ and *λ*_BA_.

## Figures and Tables

**Table 1:** Partial optimized geometric structural parameters of topic complexes in the ground and excited states associated with the experimental values of [Pt(meim)_2_]^2+^.

	[Pt(meim)_2_]^2+^				[Pt(cyim)_2_]^2+^			[Pt(meim)(cyim)]^2+^	
	Exp^10^	S_0_/^1^A_g_	T_1_/^3^B_u_	MC State/^3^B_g_	S_0_/^1^A_g_	T_1_/^3^B_u_	MC State/^3^B_g_	S_0_/^1^A′	T_1_/^3^A″
*Bond length* (Å)									
*r* (Pt–C1)	2.026	2.032	2.042	2.292	2.034	2.051	2.293	2.031/2.036	2.036/2.058
*r* (C1–N1)	1.345	1.353	1.378	1.357	1.355	1.382	1.357	1.352/1.354	1.389/1.370
*r* (C1–N2)	1.352	1.361	1.384	1.365	1.364	1.381	1.366	1.363/1.364	1.395/1.376
*r* (N2–C4)	1.454	1.458	1.451	1.458	1.458	1.450	1.457	1.455/1.458	1.449/1.453
*Bite angle* (degree)									
*a* (C1–Pt–C7)	84.2	83.3	84.5	81.6	82.9	84.0	81.0	83.1/83.0	83.8/84.0
*a* (C1–Pt–C7a)	95.9	96.7	95.5	98.4	97.1	95.8	99.0	96.5/96.6	96.1/96.1
*a* (N1–C1–Pt)	133.0	134.2	133.2	134.6	134.7	134.2	134.9	133.9/134.7	132.9/134.1
*a* (N2–C1–Pt)	122.2	121.1	122.7	120.2	120.0	121.3	119.2	121.2/120.1	123.1/121.2
*a* (N1–C1–N2)	104.8	104.6	103.8	104.0	105.0	104.1	104.4	104.7/105.0	103.5/104.4
*Dihedral angle* (degree)									
*d* (C7–C1–Pt–C7a)	180.0	180.0	180.0	180.0	180.0	180.0	180.0	179.6	178.2
*d* (C1–C4–Pt–C7)	124.7	122.2	125.9	126.6	118.4	122.7	123.7	122.0/121.1	124.7/123.0

**Table 2:** The metal d orbital components in the frontier orbitals of [Pt(cyim)_2_]^2+^ and [Pt(meim)_2_]^2+^ in different states and MC state reorganization energies (λ_(d-d)_) and inner reorganization energies (λ_i(h)_ and λ_i(e)_) (h: hole; e: electron). (HSOMO: the highest singly occupied molecular orbital).

		[Pt(meim)_2_]^2+^	[Pt(cyim)_2_]^2+^	TPD [36]
S_0_	LUMO	0.2%	0.3%	
	HOMO	35.3%	33.1%	
T_1-MLCT_	HSOMO	0.3%	0.1%	
	HSOMO-1	20.9%	19.8%	
T_1-dd_	HSOMO	34.2%	31.3%	
	HSOMO-1	10.3%	8.7%	
λ_(d-d)_/eV		1.456	1.419	
λ_i(h)_/eV		0.127	0.140	0.270
λ_i(e)_/eV		0.326	0.284	0.690

## Data Availability

Data are contained within the article and Supplementary Materials.

## Supplementary Materials

The following supporting information can be downloaded at: https://www.mdpi.com/article/10.3390/molecules29020524/s1, Table S1. Molecular orbital compositions (%) in the ground state for [Pt(meim)_2_]^2+^. Table S2. Absorptions of [Pt(meim)_2_]^2+^ according to the TD-DFT calculations. Table S3. Molecular orbital compositions (%) in the lowest-lying triplet excited state for [Pt(meim)_2_]^2+^. Table S4. Molecular orbital compositions (%) in the dd excited state for [Pt(meim)_2_]^2+^. Figure S1. Several important molecular orbitals of [Pt(meim)_2_]^2+^ in the ground state. Figure S2. Potential energy curves of ground state (S0) and the lowest triplet state (T1) involving the spin density. Figure S3. The HSOMO and HSOMO-1 molecular orbitals of [Pt(meim)_2_]^2+^ and [pt(cyim)_2_]^2+^ in the T1-dd state.
