# Supplementary material for: Density Functional Calculation and Evaluation of the Spectroscopic Properties and Luminescent Material Application Potential of the N-Heterocyclic Platinum(II) Tetracarbene Complexes
Source: Molecules. 2024 Jan 20;29(2):524. doi: 10.3390/molecules29020524 (PMC10820303; doi:10.3390/molecules29020524)
Supplement: Supplementary file 1 [file molecules-29-00524-s001.zip › molecules-2799555-supplementary.pdf]

Supplementary Material (ESI)

Density Functional Calculation and Evaluation of the Spectroscopic Properties and Luminescent Material Application Potential of the N-Heterocyclic Platinum(II) Tetracarbene Complexes

Bao-Hui Xia [1], Yin-Si Ma [1] and Fu-Quan Bai [1,2,*]

1 International Joint Research Laboratory of Nano-Micro Architecture Chemistry, College of Chemistry,
Jilin University, Changchun 130023, China; w_lei10@mails.jlu.edu.cn (B.-H.X.); yangyong07@mails.jlu.edu.cn (Y.-S.M.)

2 State Key Laboratory of Luminescent Materials and Devices, South China University of Technology,
Guangzhou 510640, China

* Correspondence: baifq@jlu.edu.cn; Tel.: +86-431-88498966

THE TABLE OF CONTENTS

Table S1 Molecular orbital compositions (%) in the ground state for $[Pt(meim)_2]^{2+}$………….. 2

Table S2 Absorptions of $[Pt(meim)_2]^{2+}$ according to the TD-DFT calculations. ……………….. 2

Table S3 Molecular orbital compositions (%) in the lowest-lying triplet excited state for $[Pt(meim)_2]^{2+}$…………………………………………………………………………………. 3

Table S4 Molecular orbital compositions (%) in the dd excited state for $[Pt(meim)_2]^{2+}$………. 3

Fig. S1 Several important molecular orbitals of $[Pt(meim)_2]^{2+}$ in the ground state……………... 4

Fig. S2 Potential energy curves of ground state (S_0) and the lowest triplet state (T_1) involving the spin density……………………………………………………………………….. 4

Fig. S3 The HSOMO and HSOMO-1 molecular orbitals of $[Pt(meim)_2]^{2+}$ and $[pt(cyim)_2]^{2+}$ in the T1-dd state……………………………………………………………………….. 5

Table S1. Molecular orbital compositions (%) in the ground state for $[Pt(meim)_2]^{2+}$.

Orbital	Energy (eV)	Orbital populations (%)							Bond type
		Pt						N-Carbene	
		$\Sigma_{,s,p}$	d_{z^2}	d_{xz}	d_{yz}	$d_{x^2-y^2}$	d_{xy}		
107 ($31a_g$)	-5.1688	0.1	0.2		0.2		0.2	99.4	π*
106 ($24b_g$)	-5.3634			0.3		15.3		83.7	$\pi^*+d_{x^2-y^2}$
105 ($24a_u$)	-5.5544	1.0						98.6	π*
104 ($28b_u$)	-5.7101	0.2	1.3	3.2	4.4	0.2	4.4	90.7	π*
103 ($27b_u$)	-7.2279	20.0 (p)						79.8	π*+p
				HOMO−LUMO Energy Gap					
102 ($30a_g$)	-12.1135	0.2	1.5	11.2	20.2	1.1	0.1	65.7	$\pi+d_{yz}+d_{xz}$
101 ($23b_g$)	-12.1620				1.3	0.3	24.4	74.0	$\pi+d_{xy}$
100 ($23a_u$)	-12.5377	0.3						99.6	π
99 ($28a_g$)	-12.6983	14.2 (s)	55.8	18.6	1.6	0.2		5.6	d_{z^2}, s, p
96 ($22b_g$)	-13.7647	1.4						98.6	π

Table S2. Absorptions of $[Pt(meim)_2]^{2+}$ according to the TD-DFT calculations. *f*: oscillator strength.

Transition		$\psi_o \rightarrow \psi_v$ (CI coeff)	E_{ver},nm(eV)	*f*	Assignment
$X^1Ag \rightarrow A^1Bu$	H→L	$30a_g \rightarrow 27b_u$ (0.69)	302.2 (4.10)	0.2182	MLCT/π→π*
$X^1Ag \rightarrow B^1Au$	H-1→L	$23b_g \rightarrow 27b_u$ (0.69)	294.6 (4.21)	0.1139	MLCT/π→π*
$X^1Ag \rightarrow C^1Bu$	H-3→L+1	$28a_g \rightarrow 28b_u$ (0.68)	271.7 (4.56)	0.1444	MLCT
$X^1Ag \rightarrow D^1Au$	H→L+2	$30a_g \rightarrow 24a_u$ (0.68)	217.8 (5.70)	0.1238	π→π*/MLCT
$X^1Ag \rightarrow E^1Bu$	H-1→L+2	$23b_g \rightarrow 24a_u$ (0.68)	210.0 (5.90)	0.1906	π→π*
	H-6→L+2	$22b_g \rightarrow 24a_u$ (0.10)			

Table S3. Molecular orbital compositions (%) in the lowest-lying triplet excited state for $[Pt(meim)_2]^{2+}$.

α				β			
Orbital	Energy (eV)	Orbital populations (%)		Orbital	Energy (eV)	Orbital populations (%)	
		Pt	N-Carbene			Pt	N-Carbene
107 ($31a_g$)--V	-5.3648		99.4	105 ($30a_g$)--V	-5.5569		89.9
106 ($24b_g$)--V	-5.7686	9.0 ($d_{x^2-y^2}$)	87.8	104 ($24a_u$)--V	-5.5626		99.1
105 ($24a_u$)--V	-5.9261		99.3	103 ($28b_u$)--V	-7.2938	18.1 (p)	81.7
104 ($30a_g$)--V	-6.1422		90.4	102 ($29a_g$)--V	-11.0743	22.5 (d_{yz})	65.0
HOMO−LUMO Energy Gap				HOMO−LUMO Energy Gap			
103 ($28b_u$)--O	-8.5167	20.0 (p)	80.6	101 ($23b_g$)--O	-11.9244	17.2 (d_{xy})	81.9
102 ($23b_g$)--O	-12.3277	19.8 (d_{xy})	79.1	100 ($23a_u$)--O	-12.2400		99.6
101 ($29a_g$)--O	-12.3938	15.2 (d_{yz})	74.3	99 ($27b_u$)--O	-12.5328		98.0
100 ($23a_u$)--O	-12.6877		99.6	98 ($28a_g$)--O	-12.9320	55.4 (d_{z^2}); 22.8 (d_{xz})	6.1

Table S4. Molecular orbital compositions (%) in the dd excited state for $[Pt(meim)_2]^{2+}$.

α				β			
Orbital	Energy (eV)	Orbital populations (%)		Orbital	Energy (eV)	Orbital populations (%)	
		Pt	N-Carbene			Pt	N-Carbene
107 ($25b_g$)--V	-5.1199		96.8	105 ($30a_g$)--V	-5.7030		93.2
106 ($24a_u$)--V	-5.4113		97.0	104 ($24b_g$)--V	-6.4758	29.3 ($d_{x^2-y^2}$)	70.1
105 ($30a_g$)--V	-5.9683		93.7	103 ($28b_u$)--V	-6.9928	24.2 (p)	75.1
104 ($28b_u$)--V	-7.1164	24.8 (p)	75.1	102 ($29a_g$)--V	-10.6161	38.5 (d_{xz}); 21.5 (d_{yz})	20.2
HOMO−LUMO Energy Gap				HOMO−LUMO Energy Gap			
103 ($24b_g$)--O	-9.1592	32.2 ($d_{x^2-y^2}$)	66.1	101 ($23b_g$)--O	-12.1407	14.8 (d_{xy})	84.5
102 ($23b_g$)--O	-12.3187		89.6	100 ($23a_u$)--O	-12.3032		99.4
101 ($29a_g$)--O	-12.4033	8.0 (d_{yz})	81.6	99 ($28a_g$)--O	-12.4248	38.2 (d_{z^2})	46.7
100 ($23a_u$)--O	-12.4373		99.5	98 ($27b_u$)--O	-12.5367		98.4

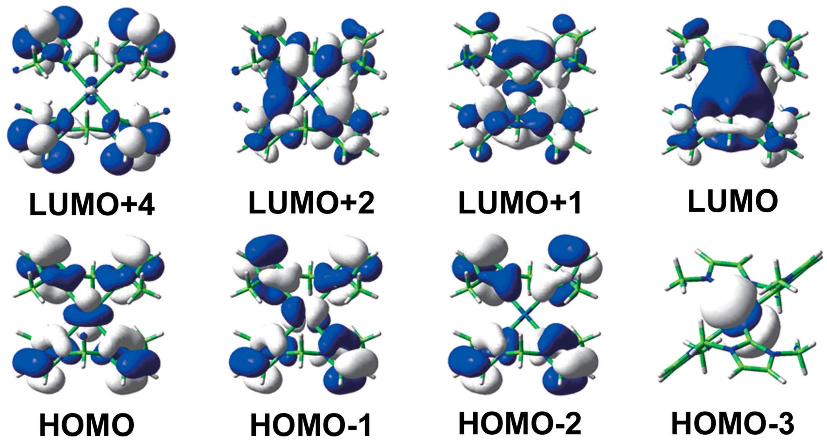

Figure S1. Several important molecular orbitals of $[Pt(meim)_2]^{2+}$ in the ground state.

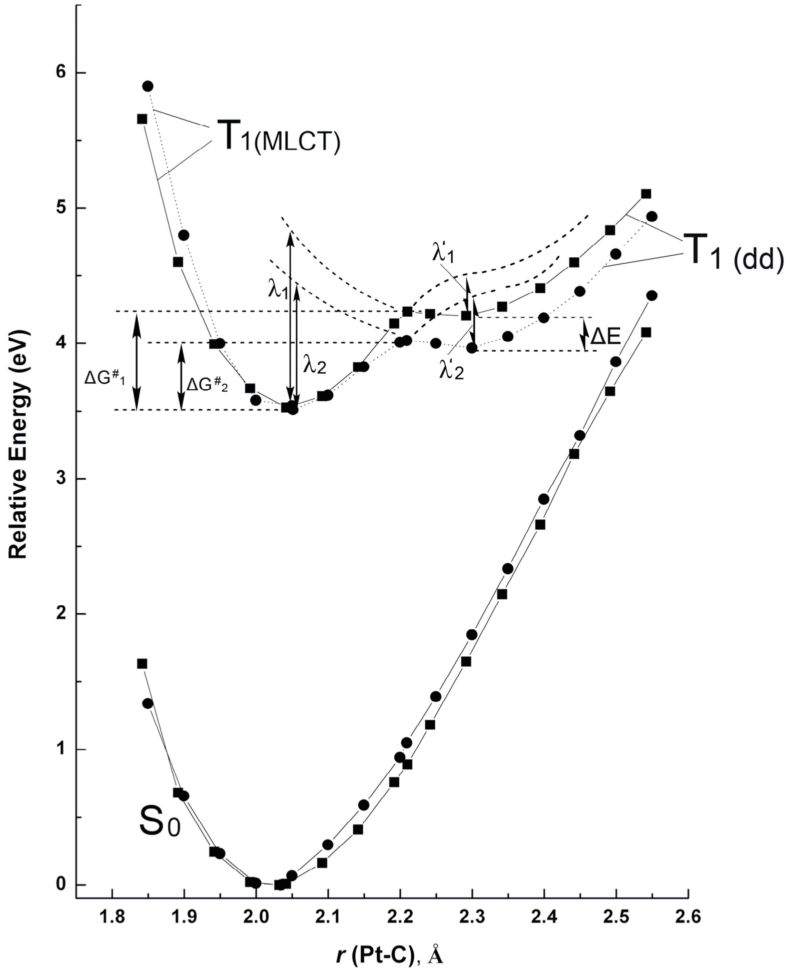

Figure S2. Potential energy curves of ground state (S_0) and the lowest triplet state (T_1) involving the spin density. (■: $[Pt(meim)_2]^{2+}$; ●: $[pt(cyim)_2]^{2+}$). Where $\Delta G^{\#}$ is the activation energy barrier, λ is the reorganization energy, and ΔE is the energy difference between two dd states. The zero-point of the energy scale is set to the global minima of the S_0-PECs, and ignore the same 0-0 transition energies. $\Delta G^{\#}_1$-$\Delta G^{\#}_2$ = 0.11 eV. ΔE = 0.24 eV.

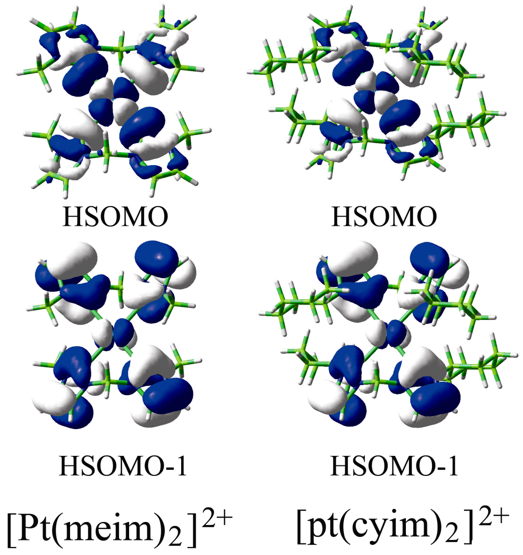

Figure S3. The HSOMO and HSOMO-1 molecular orbitals of $[Pt(meim)_2]^{2+}$ and $[pt(cyim)_2]^{2+}$ in the T1-dd state.
